# Emerging Diagnostics and Therapeutics for Non-alcoholic Fatty Liver Disease

**DOI:** 10.7759/cureus.47821

**Published:** 2023-10-27

**Authors:** Jake Darbhanga, Kiarra Krulikowski, Suzanne I Riskin

**Affiliations:** 1 Dr. Kiran C. Patel College of Osteopathic Medicine, Nova Southeastern University, Clearwater, USA

**Keywords:** nonalcoholic fatty liver, nafld treatments, nash treatments, nafld, fatty liver, nafld diagnosis, drugs for nash, nonalcoholic fatty liver disease (nafld), fatty liver disease

## Abstract

The obesity epidemic has pushed fatty liver disease, which consists of nonalcoholic fatty liver disease and nonalcoholic steatohepatitis, to the forefront of the 21st century. Disease identification can be done invasively with a liver biopsy or noninvasively through elastography and measurements of biomarkers, such as the alanine aminotransferase (ALT) and aspartate aminotransferase (AST) liver enzymes. Presently, there are no FDA-approved drugs on the market to treat the disease. Alternative medicinal treatments have been investigated, which include altering the intestinal microbiota and consuming anti-inflammatory, herbal-based, vitamin-based, and plant-based medications, in addition to following a healthy lifestyle. In this study, multiple databases were used to identify articles pertaining to fatty liver disease (FLD). Databases included Biomedical Reference Collection: Comprehensive, Cumulative Index of Nursing and Allied Health (CINAHL), Google Scholar, and PubMed. All articles gathered from the databases were peer-reviewed and less than 10 years old to ensure the credibility of the work and recent information regarding the disease. A total of 13 articles were used to gather information for this review. All articles were confirmed to be peer-reviewed by checking them with Ulrich’s web. In all 13 peer-reviewed articles, the diagnosis of FLD was most commonly done by analyzing ALT and AST liver enzymes and lipid profiles. Liver ultrasound, liver FibroScan, and liver biopsy served as other tools used for detecting the presence of FLD. It was observed that anti-inflammatory, herbal-based, vitamin-based, and plant-based medications and healthy gut microbiota had beneficial and therapeutic effects in treating FLD when coupled with healthy lifestyle changes. All medicinal treatments were found to lower the ALT and AST liver enzymes, lipid profiles (total cholesterol, triglycerides, low-density lipoprotein), and liver steatosis scores in studies where ultrasound was used before and after treatment. Further investigation is needed to fully understand the mechanisms behind the therapeutic effects of treating FLD; however, the medicinal treatments discussed in this review show promising prospects for treating the disease. The therapeutic effects of anti-inflammatory, herbal-based, vitamin-based, and plant-based medications and living a healthy lifestyle were seen in lower levels of liver enzymes, improved lipid profiles, and lower steatosis scores, with no reported side effects on subjects. The treatment options studied may have beneficial impacts in treating FLD patients and may be used in the development of future medications to combat the disease.

## Introduction and background

As the world population continues to grow and develop, more diseases and illnesses are being discovered at an alarming rate. One disease that has gained the attention of many researchers and physicians in recent years is fatty liver disease (FLD). The topic of fatty liver can be narrowed down to two disease types, namely, nonalcoholic fatty liver disease (NAFLD) and nonalcoholic steatohepatitis (NASH). NAFLD is defined as the existence of hepatic steatosis in patients not consuming excessive amounts of alcohol [1]. NASH is seen in patients who have had NAFLD for an extended time. NASH patients have steatohepatitis, otherwise known as inflammation of the liver due to the accumulation of fat in hepatocytes [1]. NAFLD has a strong association with metabolic syndrome, more specifically, obesity, which is an ever-growing concern in modern society. When NAFLD progresses to NASH, patients are at elevated risk of life-threatening conditions such as liver cirrhosis or hepatocellular carcinoma [1]. FLD is commonly diagnosed incidentally when working up other conditions that involve inspecting the liver through imaging or labs. Furthermore, symptoms that may lead a provider to suspect FLD include jaundice, weakness, pruritus, and nausea, while the patient may present as obese on a physical examination [1]. The present issue is that many primary care physicians lack a standard treatment method, which can lead to disease progression discrepancies between different practices. There is no FDA-approved medication that specifically targets FLD. This highlights the importance of exploring alternative therapeutic options to address FLD. A clear and concise diagnostic method is crucial for identifying FLD early and enabling the prompt use of emerging therapeutic methods to manage and potentially reverse the disease.

## Review

Search strategy

The search string consisted of NAFLD treatments using various spellings OR NASH treatments OR FLD treatments with peer-review and newer than 2012 as restrictions. The following databases were searched: Biomedical Reference Collection: Comprehensive and Cumulative Index of Nursing and Allied Health (CINAHL). The search yielded 832 articles. After the deletion of duplicates, 479 articles remained. Titles and abstracts were reviewed for the mention of diagnosis and treatments for NAFLD and NASH, which led to 460 articles being excluded. Full-text review of 19 articles resulted in 11 articles being included in the review. Included articles investigated diagnostic methods of FLD or prospective treatment options. Seven articles were randomized controlled clinical trials of potential FLD treatments. Inclusion criteria consisted of studies using aspartate aminotransferase (AST) and alanine aminotransferase (ALT) liver enzymes as the primary method for diagnosing FLD and monitoring treatment or ultrasound used to diagnose and monitor the treatment of FLD. Clinical trial articles were excluded if they were not randomized controlled studies. Two articles were gathered from PubMed through Google Scholar by direct search using the terms “fatty liver in primary care” and “how fibroscan works.” A total of 13 articles were included in this review. All articles were confirmed to be peer-reviewed by checking them with Ulrich’s web.

Current methods of diagnosis

The diagnosis of hepatic steatosis is most commonly done through imaging of the abdominal region. Ultrasound can be used to detect fat in the liver and is commonly used to assist in the diagnostic process. The FibroScan is a special ultrasound tool that can provide details regarding fibrosis in the liver. The FibroScan measures shear wave velocity and converts this measurement into a kilopascal score of liver stiffness. It also reports steatosis, graded by a given controlled attenuation parameter (CAP) score in decibels per meter (dB/m) [2-4]. There are three grades for liver steatosis based on the CAP score, i.e., grade S1 steatosis, which has 11-33% fatty change and a CAP score between 238 and 260 dB/m, grade S2 steatosis, which has 34-66% fatty change and a CAP score between 260 and 290 dB/m, and grade S3 steatosis, which has >67% fatty change and a CAP score >290 dB/m [2]. If a patient can be graded on the CAP score, then this is diagnostic for FLD. In addition to using ultrasound-based techniques to determine FLD, the United States Fatty Liver Index (USFLI) serves as another noninvasive method to diagnose FLD by considering ethnicity, age, gamma-glutamyl transpeptidase (GGT), waist circumference, fasting insulin, and fasting glucose levels to give a score between 0 and 100. A score >60 is representative of fatty liver while a score <30 excludes NAFLD [1,5]. In addition to the USFLI, the NAFLD fibrosis score (NFS) is another proven and reliable method for identifying advanced fibrosis as a result of FLD by using albumin measurement, platelet count, AST, age, body mass index (BMI), and fasting glucose measurements [6]. An NFS score greater than 0.676 implies that the patient most likely has advanced fibrosis while an NFS score less than -1.455 implies that there is likely an absence of advanced fibrosis [6]. Although the noninvasive measurements are reliable, the gold standard in the diagnosis of disease advancement, steatosis grade, and fibrosis stage is liver biopsy [1,3]. Liver biopsies are often avoided due to the high cost, invasiveness, and risk of potential sampling error [1]. These diagnostic methods are reliable sources for diagnosis and FLD disease progression; although ALT and AST have been noted by the American Association for the Study of Liver Diseases as reliable noninvasive markers used to monitor and diagnose hepatic dysfunction and are commonly used as an indicator for physicians to make quick inferences about a patient’s liver [7].

Emerging treatments for FLD

Treatment for FLD is less complicated in comparison to the several techniques used to diagnose the disease and determine its advancement. Currently, there is no FDA-approved medication to treat FLD. The standard treatment for FLD recognized by physicians and researchers is making lifestyle modifications, such as eating healthy and living an active lifestyle with moderate exercise [1,6]. Medicinal treatments being prescribed by physicians to treat FLD include metformin, vitamin E, silymarin (milk thistle), and pioglitazone [8]. Modern emerging treatments for FLD to be explored include altering the gut bacteria, consuming anti-inflammatory drugs such as hesperidin, consuming cholesterol-reducing drugs such as statins, use of artichoke leaf extract (ALE), fenofibrate, and potentially combining one or more of these treatment methods to yield a synergistic treatment.

**Figure 1:**
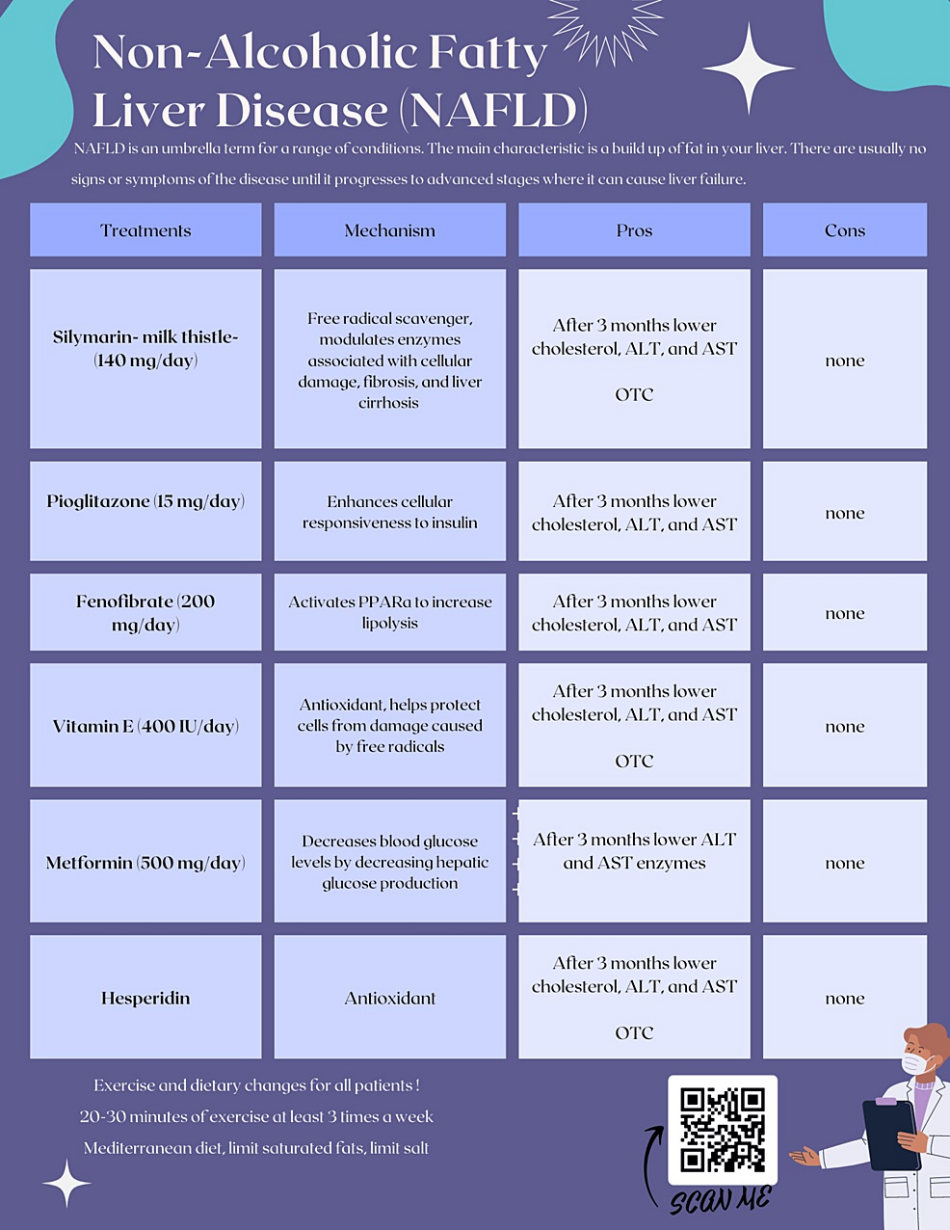
Treatment options for nonalcoholic fatty liver disease in the primary care setting.

As there is no standard of treatment for FLD aside from exercise and diet, many of the treatments mentioned above may be applicable in a primary care setting. However, primary care physicians may find it difficult to determine the best off-label treatment for each patient due to the numerous possible options. To help with this issue, a concise pamphlet consisting of potential treatment options for NAFLD is shown in Figure 1. This pamphlet can be used by primary care physicians in their offices as a reference for potential NAFLD treatments.

Hesperidin is a bioflavonoid commonly found in citrus products. Studies have predicted that the anti-inflammatory aspects of hesperidin can improve NAFLD biomarkers. Participants were told to take two 500 mg of the pills given to them every day for 12 weeks in addition to following a healthy lifestyle, which included exercising for 30 minutes three times a week and eating a healthy diet [9]. The results of this study showed that all inflammatory markers including the ALT liver enzyme, AST liver enzymes, gamma glutamyltransferase, fasting blood sugar, triglyceride, total cholesterol, low-density lipoprotein (LDL), high-sensitivity C-reactive protein, tumor necrosis factor-alpha, and Nuclear factor kappa B were significantly less in the hesperidin group relative to the control group. Furthermore, high-density lipoprotein (HDL) levels were significantly higher in the hesperidin group, which demonstrates improvement in blood lipid content as higher HDL is associated with being in better health while lower LDL is associated with being in better health. Lastly, the liver steatosis score was significantly less in the hesperidin group, as measured by the FibroScan, after 12 weeks of treatment. In the 12 weeks of treatment, the patients did not report any side effects [9].

Effects similar to what the researchers found with hesperidin were seen in another study comparing placebo pills, 400 IU/day of vitamin E, 15 mg/day pioglitazone, 500 mg/day of metformin, and 140 mg/day of silymarin (milk thistle) [8]. Much like the study by Cheraghpour et al. on hesperidin, AST, ALT, cholesterol, triglyceride, HDL, LDL, and fasting blood sugar levels before and after treatment to serve as biochemical markers representing how well each treatment worked [9]. The treatment lasted for three months and patients came into the clinic once a month to have their pills counted to ensure compliance. Secondary outcomes, which included BMI and waist circumference, improved for all treatments. Furthermore, for the primary outcomes, AST, ALT, and total cholesterol levels were statically significant and lower in groups treated with pioglitazone, metformin, and silymarin (milk thistle) when compared to the control/ placebo [8].

The effects of pioglitazone on FLD treatment were explored by Yaghoubi et al. who analyzed the therapeutic effects of fenofibrate and pioglitazone on 90 NAFLD patients over the course of 12 weeks [10]. A total of 90 subjects were randomly divided into three groups consisting of 30 subjects per group. Group one was treated with dietary modifications and exercise, group two was treated with dietary modifications and exercise in addition to 30 mg/day of pioglitazone, and group three was treated with exercise and dietary modifications in addition to 200 mg/day of fenofibrate. In the beginning, eight weeks, and 12 weeks into the study, BMI and blood pressure were recorded in addition to blood tests that were used to analyze AST, ALT, triglycerides, total cholesterol, LDL, HDL, and blood sugar levels. Researchers observed that BMI, blood pressure, ALT, and AST liver enzymes were significantly lower post-intervention in the two intervention groups, i.e., group two and group three. Furthermore, it was observed that subjects taking pioglitazone had decreased AST and ALT relative to the fenofibrate group. Subjects taking fenofibrate also had lower lipid profiles (triglycerides and total cholesterol) relative to those taking pioglitazone. These results demonstrated the therapeutic effect of fenofibrate and pioglitazone in treating NAFLD [10].

Similar to the bioflavonoid hesperidin, the effect of ALE on 89 NAFLD patients with steatosis grades between 1 and 3 was analyzed by Panahi et al. A total of 49 patients were randomly placed in the intervention group that was given 200 mg tablets of ALE and was told to take them every eight hours for two months [7]. The control/placebo group consisted of 40 patients who were told to take the placebo every eight hours for two months. Both groups were told to follow healthy lifestyles. Before and after the treatment, the fasting blood sugar, glucose, insulin, total cholesterol, LDL, triglycerides, HDL, bilirubin, ALT, alkaline phosphatase, and AST were recorded to determine whether the treatment was beneficial. Furthermore, an ultrasound was used before and after treatment to determine steatosis. The researchers concluded that ALE had therapeutic effects in treating NAFLD as the ALT, AST, and bilirubin levels were lower in the intervention group compared to the control group. The liver size was smaller in the intervention group in addition to increasing hepatic flow velocity, which demonstrates the beneficial effects of ALE treatment [7].

An aspect of FLD patients that is commonly unnoticed by physicians is the gut microbiota. The gut microbiota serves as a line of defense against intestinal diseases and systemic infections. When the flora is disrupted, generally by a broad-spectrum antibiotic, patients are at a greater risk of infections. Currently, many researchers are discovering the importance of the gut microbiome not only as a line of defense against infections but also as a key player in the absorption of nutrients, which can directly influence the progression of FLD.

The above concept was explored in a study by Mouzaki et al. in which the bile acid (BA) profiles of NAFLD patients, NASH patients, and healthy controls were compared to discover differences between the composition of BA between the three groups [11]. Additionally, BA synthesis and the gut microbiota between the three groups were studied and compared to gain insight into how these characteristics vary in NAFLD and NASH patients. Ultimately, 53 subjects were selected and categorized into 25 healthy controls, 12 NAFLD, and 16 NASH patients. The patients were instructed on how to record a seven-day food diary in addition to collecting and transporting stool samples to be analyzed. The results demonstrated that the fecal BA was elevated and that the primary-to-secondary BA ratio was higher in NASH and NAFLD patients relative to the healthy controls. Additionally, cholic acid was higher in the NAFLD and NASH groups relative to the healthy controls. The study also gathered data on C4, which demonstrated elevated levels in NASH patients compared to the healthy controls. C4 is also known as 7-alpha-hydroxy-4-cholesten-3-one and is an intermediate in BA synthesis, which serves as a reliable marker for BA synthesis. The study also accumulated IM data on 18 of the healthy controls and 11 of the NASH patients. The results showed that NASH patients had significantly fewer *Bacteroidetes *spp. and *Clostridium leptum* relative to the healthy controls. Furthermore, the study determined that NASH patient’s BA profiles were altered, as seen in the high levels of cholic acid, chenodeoxycholic acid, and increased BA synthesis, as characterized by the C4 [11]. The intestinal microbiota of FLD patients was further explored in the study by Bakhshimoghaddam et al. who hypothesized that symbiotics found in yogurt can decrease hepatic steatosis and liver enzymes in NAFLD patients [12]. To test their hypothesis the researchers tested 52 women and 50 men with a mean age of 40 years who had NAFLD by dividing them into three groups of 32 people in each. Of the three groups, the control group was directed to follow a healthy lifestyle, the intervention group was directed to follow a healthy lifestyle and consume yogurt containing 108 CFUs of B*ifidobacterium animalis* subspp. *lactis *(BB-12)/mL, and 1.5 g of inulin, and the conventional group was told to follow a healthy lifestyle in addition to consuming the yogurt without the synbiotic mentioned in the intervention group. The study lasted 24 weeks, and at week zero and every eight weeks, the blood pressure, ALT, AST, GGT, alkaline phosphatase (ALP), triglycerides, LDL, HDL, and fasting blood sugar levels were measured. Significant differences between the synbiotic group and the other two groups in serum concentrations of ALT, AST, ALP, and GGT liver enzymes were noticed, with the synbiotic group having the lowest concentration of every one of the biochemical markers mentioned. Furthermore, significance was seen between the synbiotic group and the other two groups in the secondary measures, which included total cholesterol, triglycerides, and LDL. Ultimately, the researchers concluded that synbiotics present in yogurt decrease the steatosis and biomarker levels associated with NAFLD [12]. These findings combined with the findings that *Bacteroides *spp. and *Clostridium leptum *were decreased in NASH patients clearly demonstrate the importance of the gut flora in FLD patients [11,12].

The ability to control aspects of nutrient absorption and the development of lipids may play a key role in treating FLD patients. Li et al. analyzed the effects of alcohol, cholesterol, and the cholesterol inhibitor ezetimibe [13]. A total of 12 mice were divided into four groups based on weight. The mice in each group were fed either the Leiber-DeCarlie liquid diet (LD), cholesterol LD (0.5%), alcohol LD (4%), or cholesterol (0.5%) plus alcohol LD (4%). Feeding was performed for five weeks to allow FLD models to develop. Additionally, 10 rats were placed in three groups of 10 based on their weight. The normal group received a standard pellet diet and water, and the high fat-cholesterol-sucrose and alcohol rats received the high fat-cholesterol-sucrose diet and 22% alcohol for four weeks. For the remaining 12 weeks, the high fat-cholesterol-sucrose and alcohol rat group received the same diet as the previous four weeks, while the ezetimibe group received their existing diet plus ezetimibe, which inhibits absorption of intestinal acquired cholesterol. Following the experiment, the rats and mice were euthanized, and their livers were stored to be analyzed under a microscope as well as western blot analysis. Furthermore, biochemical markers included AST, ALT, triglycerides, total cholesterol, ALP, HDL, and LDL. The results demonstrated that the cholesterol plus alcohol group of mice had a significant increase in the liver enzymes AST, ALT, and ALP, serving as an indicator of potential liver damage. Regarding cholesterol, NPC1L1 expression was associated with cholesterol absorption in the small intestine. Following the experiment, NPC1L1 expression was significantly higher compared to before the start of the experiment. Ezetimibe is an inhibitor of NPC1L1 expression, and in the mice that received the inhibitor, the biomarkers for FLD were lower, which indicates that cholesterol metabolism serves as a proponent for fatty liver associated with alcohol consumption [13].

Emerging FLD treatment shows a beneficial correlation with a reduction in FLD diagnostic biomarkers

The articles analyzed share the commonality of providing methods to facilitate monitoring the progression of FLD and provide potential treatment options. In seven articles reviewed, exercise and diet were mentioned as reliable methods to combat FLD. Three of the seven articles suggested 20 to 30 minutes of exercise at least three days a week. One article specifically mentioned the Mediterranean diet as a positive change in lifestyle, while three additional articles stated diet modifications including lowering salt, saturated fat, and carbohydrates. In six of the seven articles reviewed, ALT and AST were regarded as the main biomarkers used to assist in diagnosis and monitoring treatment. The enzymes also serve to differentiate between NAFLD and alcoholic FLD as AST levels are roughly two times higher than ALT levels in alcoholic FLD. They played a role in determining whether ALE, pioglitazone, fenofibrate, hesperidin, silymarin, metformin, vitamin E, and synbiotics from yogurt had therapeutic effects in treating FLD [9-12]. Decreases in the ALT and AST liver enzyme levels in addition to a lower AST/ALT ratio, which represents an improvement in hepatic function, were present for all treatments studied. Liver steatosis has been noticed to decrease hepatic flow velocity and increase portal vein diameter, which can affect the efficiency of the liver to execute functions and increase oxidative stress [7]. Anti-inflammatory treatments, such as ALE and hesperidin, are believed to decrease oxidative stress, which can improve liver function. ALE was seen in the study by Panahi et al. to improve NAFLD patients’ liver enzymes, increase hepatic flow velocity, and decrease steatosis-induced oxidative stress, as demonstrated by a smaller liver size post-treatment [7]. Hesperidin not only improved liver biomarkers but also lowered total cholesterol, triglycerides, and LDL [9]. In addition to HDL, total cholesterol, triglycerides, and LDL were considered lipid profile markers used to monitor treatment benefits in six of the seven studies reviewed.

Lipid profile levels are closely related to the overall wellness of the liver. High cholesterol is known to induce FLD by causing oxidative stress owing to the impairment of the mitochondrial function within the liver cells, which leads to hepatic steatosis, NAFLD, and NASH [13]. Regarding cholesterol absorption mechanisms, NPC1L1 expression is important for the absorption of cholesterol in the small intestine. As seen in the study by Li et al., in both the cholesterol-rich diet by itself and the cholesterol-rich diet plus alcohol group, the NPC1L1 expression was significantly higher compared to before the start of the experiment [13]. Based on this information, the researchers incorporated an inhibitor of NPC1L1 called ezetimibe, which was shown to decrease the biomarkers and lipid profile levels [13]. Knowing that the expression of receptors such as NPC1L1 promotes intestinal cholesterol absorption, which amplifies the negative effects of FLD, future research into drugs that are associated with decreased cholesterol metabolism, such as statins, can be explored as potential treatment options for FLD. Yaghoubi et al. noticed that fenofibrate had beneficial properties in lowering the lipid profiles in NAFLD patients while pioglitazone lowered the liver enzymes [10]. Physicians may consider giving medications based on which biomarkers need the most improvement. Administration of both fenofibrate and pioglitazone was not studied but should be investigated to determine possible synergistic effects.

Alteration of the intestinal microbiota may be beneficial to treating FLD. In the study by Farnush et al., ALT, AST, ALP, and GGT were significantly lower in the group treated by the synbiotic. Furthermore, total cholesterol, triglycerides, and LDL levels were significantly lower in the synbiotic group relative to the yogurt-only and control group [12]. The mechanism behind how probiotics work in treating FLD is believed to be that a healthy microbiome can increase gut hormones such as GLP-2, which is noted to increase insulin sensitivity in hepatocytes and adipose tissue while decreasing intestinal permeability of endotoxins [12]. Altering the gut microbiome should be further researched and possibly coupled with FLD medications possessing a different mechanism and a healthy lifestyle to yield a synergistic effect.

A limitation found within the human studies is that liver biopsies were not used before and after treatments in all of the studies due to ethical reasons and the risks associated with an invasive procedure. Another limitation includes the time span of the experiments. The experiments were not performed for a sufficiently long duration to determine whether they would be safe in the long term and whether the beneficial effects from the experiment would continue as the treatment continued. This was due to the high costs and potential loss of patient follow-up associated with longitudinal studies.

## Conclusions

There are various treatment options available on the market to potentially treat FLD. Appropriate management of contributing diseases such as diabetes mellitus and obesity can decrease the risk of FLD progression. Exercise and dietary modifications should serve as the primary intervention method in treating FLD to which the treatments studied can be added for a synergistic treatment effect. All available treatments were found to improve the biomarkers associated with FLD. The best medication choice depends on specific patient needs; therefore, primary care physicians should work with patients to prescribe the optimal treatment based on their preferences and comorbidities. This study also provides primary care physicians with the necessary information that can be used to educate patients about FLD and the mechanisms behind the chosen treatments. Proper education on the topic may motivate patients to comply with their treatment and work to halt disease progression.

Researchers can use the analyzed treatments to develop treatment protocols and potentially create new drugs to treat FLD using active moieties from various compounds. Furthermore, the treatments used in the analysis showed no concerning side effects. While waiting for FDA-approved drugs to become available, the options mentioned in this review could serve as potential treatment options for FLD.
